# The effect of food on the pharmacokinetics of WXFL10203614, a potential selective JAK1 inhibitor, in healthy Chinese subjects

**DOI:** 10.3389/fphar.2022.1066895

**Published:** 2022-11-24

**Authors:** Kai Huang, Yunfei Shi, Nannan Chu, Linling Que, Ying Ding, Zhenzhong Qian, Wei Qin, Xianghong Gu, Jiakun Wang, Zhiwei Zhang, Jianguo Xu, Qing He

**Affiliations:** ^1^ Drug Clinical Trial Institution, Affiliated Wuxi People’s Hospital of Nanjing Medical University, Wuxi, China; ^2^ Wuxi Fuxin Pharmaceutical Research and Development Co., Ltd., Wuxi, China

**Keywords:** WXFL10203614, pharmacokinetics, JAK1 inhibitor, rheumatoid arthritis, absorption

## Abstract

**Objective:** This study was performed to investigate the effect of food on the pharmacokinetics (PK) of WXFL10203614 in healthy Chinese subjects.

**Methods:** This was a randomized, open-label, single-dose, two-treatment (fed vs fasted), two-period, two-sequence, crossover study. 14 eligible subjects were averagely randomized into 2 sequences and then received 10 mg WXFL10203614 under fasted or fed condition. In each period, the blood samples were collected from 0 h (pre-dose) and serially up to 72 h post-dose, and plasma concentrations were detected using the high-performance liquid chromatography-tandem mass spectrometry (HPLC-MS/MS) method. The effect of food on the PK profile and safety of WXFL10203614 were assessed.

**Results:** 70 subjects were screened, and 14 subjects (10 male and 4 female) were enrolled and completed the study. Under the fasted condition, WXFL10203614 was absorbed rapidly with a T_max_ of 0.98 h. The absorption rate was slower, T_max_ delayed by 2.98 h, and the C_max_ decreased by 16.3% when WXFL10203614 administered after the high-fat and high-calorie diet, other PK parameters were not affected. The 90% confidence intervals (CIs) for the ratio (fed/fasted) of geometric means of the C_max_, AUC_0-t_ and AUC_0-∞_ were 0.73–1.01, 0.90–1.03 and 0.90–1.03, indicating that the high-fat and high-calorie diet might impact the absorption process of WXFL10203614. Although the C_max_ was slightly decreased, there was no significant difference in the C_max_ under fasted and fed conditions. Thus, it was not considered clinically significant owing to the small magnitude of changes in C_max_. All Treatment-emergent adverse events (TEAEs) were mild and resolved spontaneously without treatment.

**Conclusion:** Food had no clinically relevant effects on drug system exposure of WXFL10203614. It was well tolerated under fasted and fed conditions in healthy Chinese subjects, so WXFL10203614 could be administered orally with or without food.

**Clinical Trial Registration**: http://www.chinadrugtrials.org.cn/index.html, identifier CTR20191636.

## Introduction

Rheumatoid arthritis (RA) is an autoimmune disease associated with chronic and painful joint inflammation, which gradually causes joint synovitis, bone erosion, progressive cartilage destruction, and even physical disability (Sparks, 2019). However, the exact pathogenesis of RA is unknown (Li et al., 2020). It has been reported that Janus kinase (JAK)/signal transducers and activators of transcription (STAT) signal pathway was closely related to various inflammatory cytokines exerting and integrating their functions in inflammatory diseases, such as RA (Fragoulis et al., 2019). As the key initiators of the JAK/STAT signal pathway, JAK was increasingly regarded as one of the critical factors in the pathogenesis of RA (Crispino et al., 2021). Therefore, inhibiting the JAK/STAT signal pathway with specific JAK inhibitors is a new therapeutic option for RA.

WXFL10203614, a potential selective JAK1 inhibitor, is being developed for RA treatment. At present, WXFL10203614 has entered Phase Ⅲ clinical trial stage. As a part of the new drug application, assessing the effects of food on oral drugs was strongly recommended by guidelines (EMA, 2012; NMPA, 2021; USFDA, 2022). Food-drug interaction was often associated with changes in drug pharmacokinetics (PK), which inadvertently affected the drug effect. However, the reasons for food-drug interaction were complicated, such as the chelation of drug with components in food, the direct interactions between drug and food, delayed gastric emptying, gastric acid or bile secretion increased, and food-induced increase in drug solubility, etc. (Singh, 1999; Schmidt et al., 2002; EMA, 2012). This interaction would result in decreased, delayed, increased or accelerated drug absorption. Therefore, food-drug interaction, on the one hand, might cause a high risk of treatment failure due to a remarkably reduced systemic drug exposure. On the other hand, it might obtain the desired pharmacological effect but with severe toxicity (Schmidt et al., 2002). Great importance should be attached to this issue in the process of developing a new drug.

Although the effects of food on the PK profiles of JAK inhibitors have been reported previously, the consequences were not completely consistent. The C_max_ of INCB018424, filgotinib and upadacitinib was partly reduced by food which had little effect on AUC (Shi et al., 2011; Mohamed et al., 2017; Anderson et al., 2019). In contrast, the C_max_ of tofacitinib and peficitinib was elevated to different degrees under the fed state (Lamba et al., 2016; Shibata et al., 2021). However, it is not yet clear whether food interferes with the PK profile of WXFL10203614.

This study was conducted on healthy Chinese subjects to assess the effect of the high-calorie and high-fat diet on the PK profile of WXFL10203614. Meanwhile, safety was also evaluated.

## Methods

### Subjects

Healthy Chinese subjects, 18–45 years old, with body weight ≥50 for males and ≥45 kg for females, and body mass index (BMI) ranging from 19.0 to 26.0 kg/m^2^ were enrolled. Meanwhile, they were assessed as healthy by vital signs, physical examination, medical history, laboratory tests, abdominal ultrasound, chest X-ray and electrocardiograph (ECG). Subjects could not participate if they were confirmed to meet one of the exclusion criteria as follow: 1) the history of the cardiovascular, nervous, respiratory, digestive or other systems, 2) allergic constitution or severe allergies to any medicine or food, 3) abnormal ECG, such as QTc interval ≥450 ms, PR interval ≥210 ms or QRS ≥120 ms, 4) severe infection, 5) the positive result for hepatitis B surface antigen, hepatitis C antibody, human immunodeficiency virus (HIV) antibody or treponema pallidum antibody, 6) medicine taken within 1 month before screening (including vitamins, herbal products and dietary supplements), 7) the positive result for the urine drug test, urine nicotine test or breath alcohol test, 8) pregnant or lactating women, 9) participated in any clinical trial in the past 3 months, 10) any clinically significant abnormal findings judged by investigators, 11) other special situations confirmed by investigators for exclusion.

### Study design

This randomized, open-label, single-dose, two-treatment (fed vs fasted), two-period, two-sequence, crossover study was conducted on healthy Chinese subjects to evaluate the effect of food on the PK profile of WXFL10203614. The study was approved by National Medical Products Administration (NMPA) (Chinese Clinical Trial Registry, http://www.chinadrugtrials.org.cn/index.html, Registration Number: CTR20191636) and carried out on the basis of Good Clinical Practice guidelines and the ethical principle of the Declaration of Helsinki. The final protocol and informed consent form were reviewed and permitted by the ethics committee of the Affiliated Wuxi People’s Hospital of Nanjing Medical University (Approval Number: 2019LLPJ-I-24). All subjects were fully informed of the study procedures, objectives, basic information and potential risks of the investigational drug and provided written informed consent before enrollment.

**FIGURE 1:**
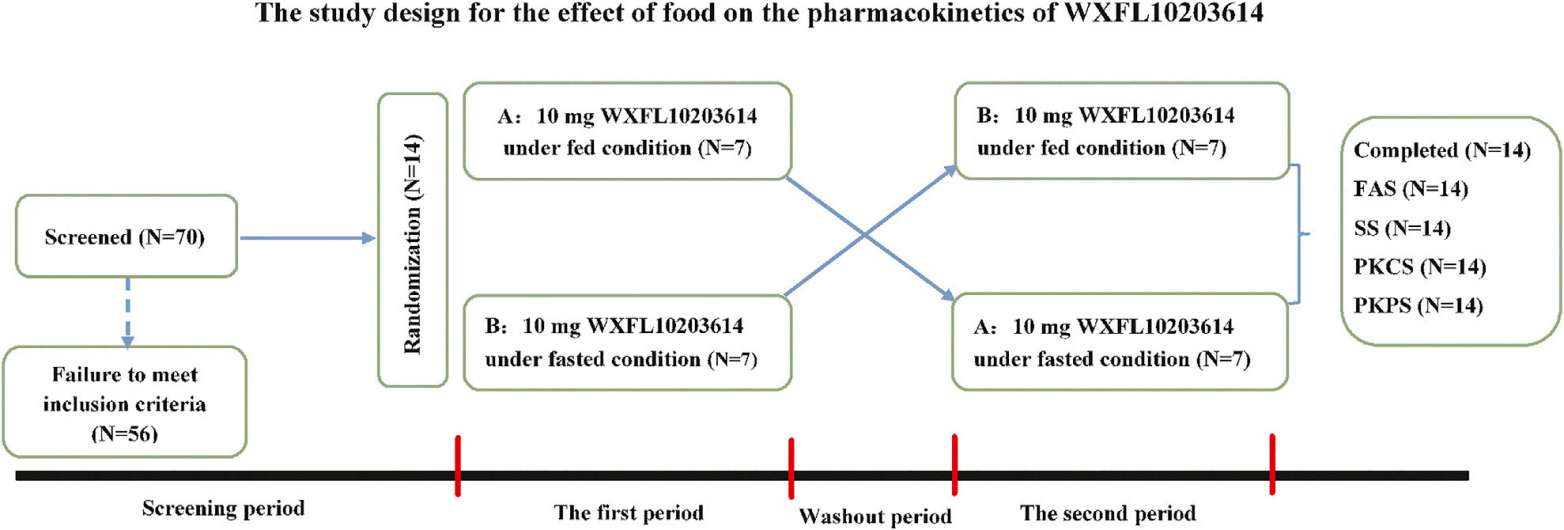
The study design for the effect of food on the pharmacokinetics of WXFL10203614. FAS, The Full Analysis Set; SS, Safety set; PKCS, Pharmacokinetic concentration set; PKPS, Pharmacokinetic parameter set.

This two-period clinical trial was conducted in the Phase Ⅰ center of Wuxi people’s hospital from August to September 2019. 14 subjects were randomly allocated into sequence A and sequence B in a 1:1 ratio, and then received a single oral dose of 10 mg WXFL10203614 (5 mg*2 tablets, Lot Number: XS180902C) with 240 ml water under fasted or fed condition with the high-fat and high-calorie diet (800–1000 calories with 150 calories from protein, 250 calories from carbohydrates and 400–600 calories from fat) (USFDA, 2022). Among them, 7 subjects in sequence A received 10 mg WXFL10203614 with food in the first period and without food in the second period, the remaining 7 subjects in sequence B received 10 mg WXFL10203614 without food in the first period and with food in the second period. The protocol design was described in detail in Figure 1. All subjects fasted overnight for at least 10 h and ate up the meal within 30 min before dosing. Moreover, none of the subjects was permitted to drink water within 1 h before and after dosing or eat anything within 4 h after dosing. All subjects were allowed to leave the clinical center on Day 3 after dosing in each period, and the washout between the 2 periods was 7 days.

### Pharmacokinetic assessment

In each period, 4 ml of blood samples were collected in EDTA-K_2_ tubes at 15 time points: 0 (pre-dose), 0.25, 0.5, 1, 1.5, 2, 3, 4, 6, 8, 12, 24, 36, 48 and 72 h following oral administration of WXFL10203614. The plasma samples were obtained after centrifuging at 1500 g for 10 min at 4°C and then stored at -80°C until analysis.

### Determination of plasma concentration of WXFL10203614

Samples were prepared by simple protein precipitation with acetonitrile. Briefly, 50 μL of internal standard (IS) solution (50 ng/ml ^13^CD_3_-Target) and 300 μL of acetonitrile were added to 50 μL of the plasma sample. The mixture was vortexed for 15 min and then centrifuged at 2510 g for 15 min. The supernates were used to determine the WXFL10203614 concentration.

The validated high-performance liquid chromatography-tandem mass spectrometry (HPLC-MS/MS) method was used to determine the concentrations of WXFL10203614 in plasma (Huang et al., 2022). The HPLC-MS/MS system consisted of the UPLC 30A system (Shimadzu, Japan) and the AB SCIEX Triple Quad 5500 system with an electrospray ionization (ESI) source and Analyst 1.6.3 software (AB SCIEX, USA) for data acquisition and processing. Multiple reaction monitoring (MRM) transitions of WXFL10203614 and ^13^CD_3_-Target (IS) were m/z 294.1→146.0 and 298.2→146.1 for quantification, respectively. Additionally, 1 μL sample was injected into a BEH C18 column (1.7 μm, 2.1 mm × 50 mm, Waters, USA) to chromatograph WXFL10203614 and IS with the gradient elution. The calibration of WXFL10203614 ranged from 0.4 to 500 ng/ml with the lower limit of quantitation (LLOQ) of 0.4 ng/ml. The accuracy of the assay was -4.2%–2.9%, and the precision was within the 6.1% coefficient of variation. Additionally, Samples were stable in blood for 2 h at room temperature and in plasma at 4°C for 17 h and -20°C and -80°C for 105 days. The 10-fold dilutions did not affect the accuracy and precision of the samples.

### Safety assessment

Treatment-emergent adverse events (TEAEs) were defined and graded according to the National Cancer Institute-Common Terminology Criteria for Adverse Events (NCI-CTCAE) (version 5.03). The severity, duration and clinical outcome of TEAEs and their association with WXFL10203614 were all evaluated. In addition, any clinically significant changes in physical examination, vital signs, laboratory tests as well as ECG were all included in the safety assessment.

### Statistical analysis

Descriptive statistics were calculated for Pharmacokinetic (PK) parameters and demographical data. The primary PK parameters were maximum concentration (C_max_), time of maximum concentration (T_max_), area under the plasma concentration-time curve from time 0 to the last measurable concentration (AUC_0-t_), area under the plasma concentration-time curve from time 0 to infinity (AUC_0-∞_), terminal elimination half-life (t_1/2_), mean residence time of 0 to the last measurable concentration (MRT_0-t_), and apparent clearance rate (CL/F) analyzed by the non-compartmental method with the WinNonlin software (version 8.2). The PK parameters were summarized descriptively. Variance analysis was used to compare the log-transformed PK parameters (C_max_, AUC_0-t_ and AUC_0-∞_) between the fed and fasted treatment groups. If the 90% confidence intervals (CIs) for the ratio (fed/fasted) of geometric means of the C_max_, AUC_0-t_ and AUC_0-∞_ fell within 0.80–1.25, confirming that food has no significant effect on the PK profile of WXFL10203614. Variance analysis and *t*-test for normally distributed data and Wilcoxon rank test for non-normally distributed data were used for data analysis. All statistical analyses were conducted with SAS software (version 9.4) and *p* < 0.05 was considered statistically significant.

## Results

### Demographic data of subjects

70 subjects were screened and 56 failed to meet the inclusion criteria. Finally, 14 eligible healthy subjects (10 male and 4 female) were randomized into 2 sequences to receive 10 mg WXFL10203614 orally under fed or fasted condition. All subjects completed the study and were included in the full analysis set (FAS), safety set (SS), pharmacokinetic concentration set (PKCS) and pharmacokinetic parameter set (PKPS) (Figure 1). Baseline demographic characteristics were summarized in Table 1 using descriptive statistics. There were no significant differences in age, height, weight and BMI between the 2 groups.

**TABLE 1:** Demographics and baseline characteristics of subjects.

Characteristic	Sequence A (N = 7)	Sequence B (N = 7)
Sex, N (%)		
Male	5 (71.4)	5 (71.4)
Female	2 (28.6)	2 (28.6)
Ethnicity (Han/other)	6/1	6/1
Age (years)	29.3 ± 5.8	25.3 ± 6.7
Height (cm)	165.0 ± 9.0	161.9 ± 7.5
Weight (kg)	61.7 ± 8.2	60.2 ± 9.7
BMI (kg/m^2^)	22.6 ± 1.0	22.9 ± 2.2

N, number; BMI, body mass index. Data are presented as mean ± standard deviation unless otherwise indicated.

### Pharmacokinetic and statistical analyses

**FIGURE 2:**
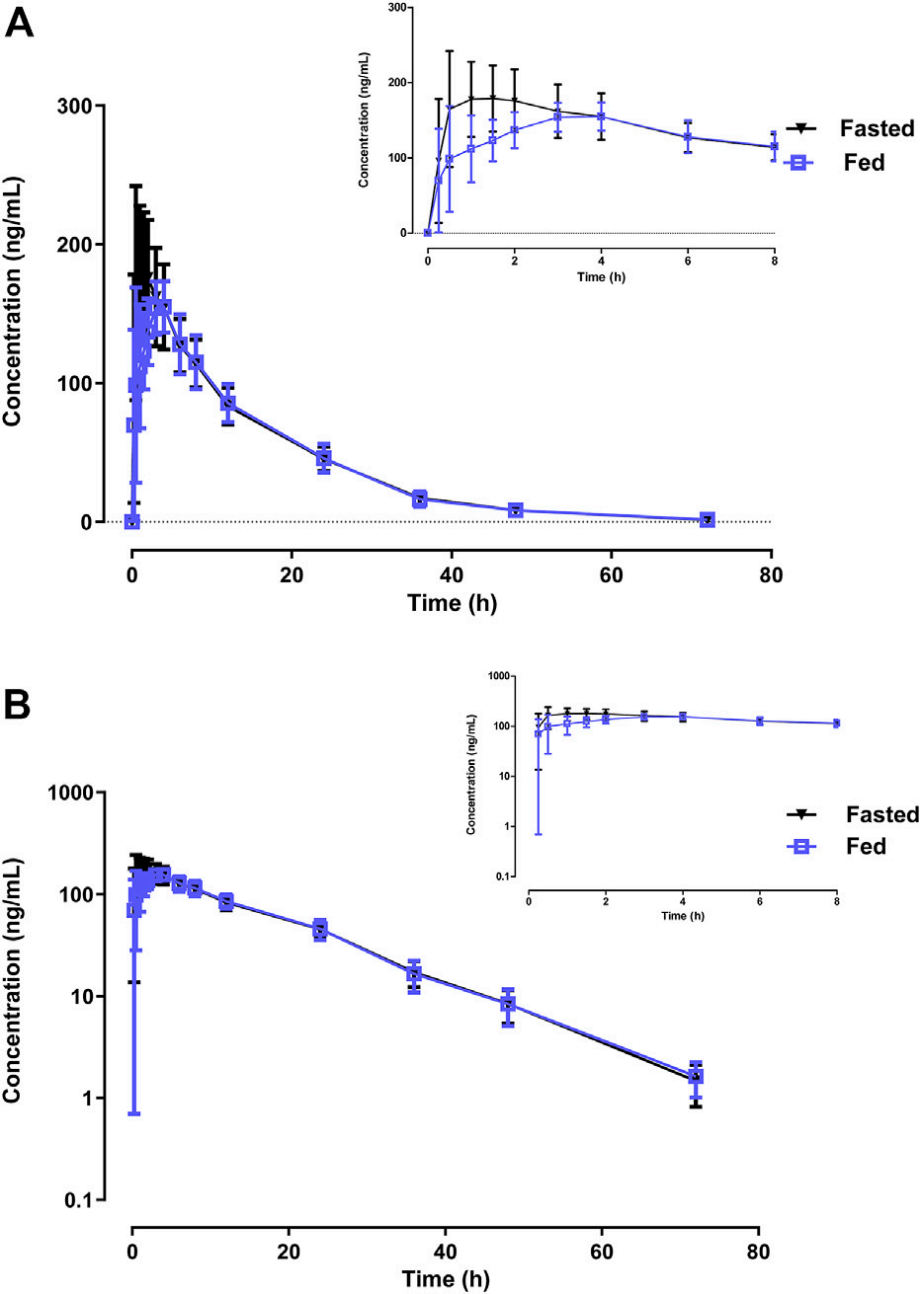
The linear **(A)** and semi-logarithmic **(B)** mean plasma concentrations-time profiles after a single dose of 10 mg WXFL10203614 under fed and fasted conditions. The data are expressed as mean ± standard deviation.

The effect of food on the primary PK parameters and mean plasma concentration-time profile of 10 mg WXFL10203614 were shown in Table 2 and Figure 2, respectively. Under the fasted condition, WXFL10203614 was absorbed rapidly with a median T_max_ of 0.98 h, but the absorption rate was delayed and T_max_ was 2.98 h, C_max_ was decreased by 16.3% after food intake (from 203.0 ± 55.3 ng/ml to 170.0 ± 29.8 ng/ml). Although the 90% CIs for the geometric mean ratios (GMRs) of the C_max_, AUC_0-t_ and AUC_0–∞_ were 0.73–1.01, 0.90–1.03 and 0.90–1.03 (Table 3), confirming that the C_max_ was beyond the acceptable range of 0.80–1.25, there was no significant difference in these PK parameters (C_max_, AUC_0-t_, AUC_0-∞,_ t_1/2_, MRT_0–t_ and CL/F) between the 2 groups (*p* > 0.05). In view of the change in the C_max_ was slight, thus it was indicated that food intake delayed the absorption of WXFL10203614 to a certain extent, but did not significantly influence the PK profiles (C_max_, AUC_0-t_ and AUC_0-∞_) of the WXFL10203614 under the fasted and fed conditions.

**TABLE 2:** The primary pharmacokinetic parameters of WXFL10203614 after dosing under fed and fasted conditions.

Parameter	Fed condition (N = 14)	Fasted condition (N = 14)
C_max_ (ng/ml)	170.0 ± 29.8	203.0 ± 55.3
T_max_ (h)	2.98 (0.23–3.98)	0.98 (0.48–3.98)
AUC_0–t_ (h⋅ng/mL)	2780 ± 453	2890 ± 467
AUC_0–∞_ (h⋅ng/mL)	2800 ± 459	2910 ± 474
t_1/2_ (h)	10.2 ± 1.3	9.9 ± 1.2
MRT_0–t_ (h)	15.2 ± 1.6	14.8 ± 1.6
CL/F (L/h)	3.66 ± 0.61	3.53 ± 0.61

C_max_, maximum concentration; T_max_, time of maximum concentration; AUC_0-t_, area under the plasma concentration-time curve from time 0 to the last measurable concentration; AUC_0-∞_, area under the plasma concentration-time curve from time 0 to infinity; t_1/2_, terminal elimination half-life; MRT_0-t_, mean residence time of 0 to the last measurable concentration and CL/F, apparent clearance rate. T_max_ is presented as median (minimum, maximum) and other data are presented as mean ± standard deviation.

**TABLE 3:** Statistical comparison of primary pharmacokinetic parameters of WXFL10203614.

Parameter	Geometric mean		Fed/fasted ratio (%)	90% CI of the Fed/Fasted ratio	Intra-CV
	Fed condition (N = 14)	Fasted condition (N = 14)			
C_max_ (ng/ml)	167.3	195.2	85.7	0.73–1.01	0.250
AUC_0–t_ (h⋅ng/mL)	2741.8	2852.1	96.1	0.90–1.03	0.098
AUC_0–∞_ (h⋅ng/mL)	2767.8	2873.9	96.3	0.90–1.03	0.099

The acceptance range of 90% confidence interval is 0.80–1.25. C_max_, maximum concentration; AUC_0-t_, area under the plasma concentration-time curve from time 0 to the last measurable concentration; AUC_0-∞_, area under the plasma concentration-time curve from time 0 to infinity; CI, confidence interval; CV, coefficient of variation.

### Safety analysis

10 mg WXFL10203614 was well-tolerated in the subjects. A total of 26 TEAEs occurred in 13 subjects (92.9%, 13/14) who received 10 mg WXFL10203614. 4 TEAEs reported in 2 subjects (14.3%, 2/14) were not associated with WXFL10203614 while 22 TEAEs reported in 12 subjects (85.7%, 12/14) were associated with WXFL10203614.

Under the fasted condition, 15 TEAEs occurred in 10 subjects (71.4%, 10/14) after dosing (Table 4), 14 TEAEs associated with WXFL10203614 occurred in 10 subjects (71.4%, 10/14), including polyuria (28.6%, 4/14), creatinine increased (21.4%, 3/14), fecal occult blood (positive for transferrin) (14.3%, 2/14), neutrophil count decreased (7.1%, 1/14), neutrophil count increased (7.1%, 1/14), white blood cell count increased (7.1%, 1/14) and fibrinogen decreased (7.1%, 1/14). 1 TEAE (soft tissue injury) was considered unrelated to WXFL10203614.

**TABLE 4:** Summary of treatment emergent adverse events.

System organ class preferred term	Fed condition (N = 14)	Fasted condition (N = 14)
Total TEAE n (%)	8 (57.1)	10 (71.4)
Investigations	6 (42.9)	7 (50.0)
Fecal occult blood (positive for transferrin)	1 (7.1)*	2 (14.3)
Neutrophil count decreased	0	1 (7.1)
Neutrophil count increased	0	1 (7.1)
White blood cell count increased	0	1 (7.1)
Triglycerides increased	1 (7.1)	0
Fibrinogen decreased	1 (7.1)	1 (7.1)
Creatinine increased	3 (21.4)	3 (21.4)
Renal and urinary disorders	3 (21.4)	4 (28.6)
Polyuria	3 (21.4)	4 (28.6)
Skin and subcutaneous tissue disorders	0	1 (7.1)
Soft tissue injury	0	1 (7.1)*
Gastrointestinal disorders	1 (7.1)	0
Diarrhea	1 (7.1)*	0

TEAT, treatment emergent adverse events; n, number of subjects; %, incidence of subjects reporting TEAEs. Data are reported as n (%). *: TEAEs were considered unrelated to the WXFL10203614.

Under the fed condition, 11 TEAEs occurred in 8 subjects (57.1%, 8/14) after receiving WXFL10203614 (Table 4), 8 TEAEs associated with WXFL10203614 occurred in 7 subjects (50.0%, 7/14), including polyuria (21.4%, 3/14), creatinine increased (21.4%, 3/14), triglycerides increased (7.1%, 1/14) and fibrinogen decreased (7.1%, 1/14), 3 TEAEs were unassociated with WXFL10203614, including diarrhea (7.1%, 1/14) and fecal occult blood (positive for transferrin) (7.1%, 1/14), because NO.005 subject had diarrhea, the results of stool routine tests were abnormal twice.

All AEs were mild and resolved spontaneously without any special intervention. None of the subjects was discontinued from the study due to AEs. There were no serious adverse events (SAEs) or deaths reported. At the end of the study, the investigator collected related information on all AEs observed in this trial, such as the name, severity, duration and clinical outcome of AEs and their association with WXFL10203614. According to the requirements of the Ethics Committee of Wuxi people’s hospital, these AEs should be reported within 3 months after the completion of the study. Finally, 26 AEs were submitted to the Ethics Committee as paper reports.

## Discussion

It is the first clinical trial conducted on healthy Chinese subjects to investigate the food effect on the PK profile of WXFL10203614. Generally, the food effect on drug exposure depends mainly on the biopharmaceutical properties and formulation of the drug, which may affect its efficacy (Sun et al., 2019). For this reason, the effect of food on oral drug should be explored at the early stage of drug development to make the dose recommendation in Phase II and Ⅲ studies (EMA, 2012). Thus, a randomized, open-label, single-dose, two-treatment (fed vs fasted), two-period, two-sequence, crossover study was designed in Phase Ⅰ clinical trial stage to evaluate the potential food-drug interaction. Our data showed that food delayed the absorption rate of WXFL10203614.

The design of our study is mainly based on the following considerations: Firstly, the greatest advantage of a crossover study is that the subjects act as self-controls allowing for greater biological homogeneity, which is strongly recommended by guidelines. Additionally, 7 days of washout is enough to eliminate WXFL10203614 based on the short half-life. Secondly, for safety concerns, healthy male and female subjects were enrolled, which was easier for investigators to observe AEs and the changes in clinical data after dosing. Thirdly, according to the guideline of NMPA, a minimum of 12 subjects should complete the study to statistically assess the food effects on drug (NMPA, 2021). The final sample size was set as 14 on the basis of the dropout rate of 10%. Fourthly, in our single dose-escalation study, 10 mg WXFL10203614 was intended to be the highest recommended dose for RA treatment, so the dose of WXFL10203614 used to evaluate the food effect was 10 mg. Finally and most importantly, in order to provide the most significant impact on gastrointestinal physiology, thereby affecting the PK profile of the drug maximally, a high-fat and high-calorie meal is generally recommended before dosing, and the compositions and calories of the meal should be followed the guidelines strictly (NMPA, 2021; USFDA, 2022).

In terms of PK profile, the results showed that the t_1/2_, MRT_0–t_ and CL/F of WXFL10203614 were similar under the fasted and fed conditions, indicating the elimination phase of WXFL10203614 was not influenced. Furthermore, the AUC was not obviously changed after food intake and the 90% CIs for the ratios (fed/fasted) of the AUC_0–t_ and AUC_0–∞_ were all within the range of 0.80–1.25, confirming that food did not affect the systemic exposure of WXFL10203614. However, for the absorption phase, the T_max_ was delayed approximately 3-fold and C_max_ decreased by 16.3% under the fed condition. More importantly, the 90% CI for the ratio (fed/fasted) of the C_max_ was not totally within the range of 0.80–1.25. The above results indicated that the high-fat and high-calorie diet slowed down the absorption rate of WXFL10203614, similar to the previously results reported in other selective JAK1 inhibitors. Administration of 3 mg upadacitinib immediate-release capsules after a high-fat meal, C_max_ decreased by 23%, and AUC was not impacted compared with the fasted condition (Mohamed et al., 2017). High- and low-fat meals decreased C_max_ by 20% and 11% and delayed the absorption of filgotinib, with T_max_ prolonging about 2-3 folds, but little effect on systemic exposure was observed (Anderson et al., 2019). In general, the delayed absorption or reduced absorption rate originated from the slower gastric emptying rate and/or increased gastric pH in the fed state, which exhibited a decreased C_max_ and a corresponding longer T_max_ in the PK profile (Singh, 1999). The onset of drug absorption usually occurred in the proximal bowel, which might be delayed due to a slower gastric emptying rate (Lennernäs et al., 1997). The drug delivered from the stomach to the small intestine became the rate-limiting step in drug absorption (Singh, 1999). Therefore, we speculated that the delayed absorption of WXFL10203614 is perhaps related to the slow gastric emptying rate, but the exact mechanism needs to be studied further. Although the C_max_ decreased by 16.3% and 90% CI did not fall within the range of 0.8–1.25, this slight decrease in C_max_ did not need the dose adjustment owing to the small magnitude of changes (<25%) and was not considered clinically significant (Shen et al., 2019). Therefore, WXFL10203614 could be taken with or without food. However, due to the limitation of the sample size in our study, it is hard to compare the effect of gender difference on PK profile.

Regarding safety, WXFL10203614 was well tolerated in all subjects under fed or fasted condition. The TEAEs were consistent with previous studies of WXFL10203614 and JAK inhibitors (Shi et al., 2014; Taylor et al., 2017; Choy, 2019; Li et al., 2022). All TEAEs were mild and resolved spontaneously without any intervention. Additionally, the number and incidence of TEAEs (the changes in the neutrophil count and white blood cell count) were slightly reduced under the fed condition. These AEs were associated with the JAK2 inhibition which interfered with the regulatory functions of granulocyte-macrophage colony-stimulating factor (GM-CSF) that stimulated the proliferation of granulocytes and macrophages from bone marrow precursor cells (Furuya et al., 2018). WXFL10203614 has a slight inhibition effect on JAK2, and the inhibition effect of WXFL10203614 on JAK1 was 9-fold than that on JAK2 *in vitro*. It is inferred that when the absorption process of WXFL10203614 is delayed and concentration in the blood is reduced under the fed condition, the inhibition on the JAK2 signal pathway is weakened, which might reduce the risk of adverse events. However, more samples will be needed to confirm this speculation in future studies.

## Conclusion

This study demonstrated that food could delay the absorption rate of WXFL10203614 but not significantly affect systemic exposure, which had no clinically relevant impact on the pharmacokinetics of the WXFL10203614 tablet. In addition, WXFL10203614 was well tolerated under fed or fasted condition so that it could be administered orally with or without food.

## Data Availability

The original contributions presented in the study are included in the article/Supplementary Material, further inquiries can be directed to the corresponding author.

## References

[B1] Anderson K. Zheng H. Kotecha M. Cuvin J. Scott B. Sharma S. (2019). The relative bioavailability and effects of food and acid-reducing agents on filgotinib tablets in healthy subjects. Clin. Pharmacol. Drug Dev. 8, 585–594. 10.1002/cpdd.659 30768860

[B2] Center for drug evaluation, National Medical Products Administration (2021). Technical guidelines for the food-effect studies in the development of new drugs . https://www.cde.org.cn/main/news/viewInfoCommon/4f21fc720672cf26ad0efbe0207fdced.

[B3] Choy E. H. (2019). Clinical significance of Janus Kinase inhibitor selectivity. Rheumatol. Oxf. 58, 953–962. 10.1093/rheumatology/key339 PMC6532440 30508136

[B4] Crispino N. Ciccia F. (2021). JAK/STAT pathway and nociceptive cytokine signalling in rheumatoid arthritis and psoriatic arthritis. Clin. Exp. Rheumatol. 39, 668–675. 10.55563/clinexprheumatol/e7ayu8 33200731

[B5] European medicines agency (2012). Guideline on the investigation of drug interactions. https://www.ema.europa.eu/en/documents/scientific-guideline/guideline-investigation-drug-interactions-revision-1_en.pdf.

[B6] Fragoulis G. E. McInnes I. B. Siebert S. (2019). JAK-inhibitors. New players in the field of immune-mediated diseases, beyond rheumatoid arthritis. Rheumatol. Oxf. 58, i43–i54. 10.1093/rheumatology/key276 PMC6390879 30806709

[B7] Furuya M. Y. Asano T. Sumichika Y. Sato S. Kobayashi H. Watanabe H. (2018). Tofacitinib inhibits granulocyte-macrophage colony-stimulating factor-induced NLRP3 inflammasome activation in human neutrophils. Arthritis Res. Ther. 20, 196. 10.1186/s13075-018-1685-x 30157949 PMC6116484

[B8] Huang K. Ding Y. Que L. Chu N. Shi Y. Qian Z. (2022). Safety, tolerability and pharmacokinetics of WXFL10203614 in healthy Chinese subjects: A randomized, double-blind, placebo-controlled phase Ⅰ study. Front. Pharmacol. 13, 1057949. 10.3389/fphar.2022.1057949 36408263 PMC9671933

[B9] Lamba M. Wang R. Fletcher T. Alvey C. Kushner J. 4th. Stock T. C. (2016). Extended-release once-daily formulation of tofacitinib: Evaluation of pharmacokinetics compared with immediate-release tofacitinib and impact of food. J. Clin. Pharmacol. 56, 1362–1371. 10.1002/jcph.734 26970526 PMC5113768

[B10] Lennernäs H. Fager G. (1997). Pharmacodynamics and pharmacokinetics of the HMG-CoA reductase inhibitors. Similarities and differences. Clin. Pharmacokinet. 32, 403–425. 10.2165/00003088-199732050-00005 9160173

[B11] Li N. Du S. Wang Y. Zhu X. Shu S. Men Y. (2022). Randomized, double-blinded, placebo-controlled phase I study of the pharmacokinetics, pharmacodynamics, and safety of KL130008, a novel oral JAK inhibitor, in healthy subjects. Eur. J. Pharm. Sci. 176, 106257. 10.1016/j.ejps.2022.106257 35820629

[B12] Li Z. Xu M. Li R. Zhu Z. Liu Y. Du Z. (2020). Identification of biomarkers associated with synovitis in rheumatoid arthritis by bioinformatics analyses. Biosci. Rep. 40, BSR20201713. 10.1042/BSR20201713 32840301 PMC7502692

[B13] Mohamed M. F. Jungerwirth S. Asatryan A. Jiang P. Othman A. A. (2017). Assessment of effect of CYP3A inhibition, CYP induction, OATP1B inhibition, and high-fat meal on pharmacokinetics of the JAK1 inhibitor upadacitinib. Br. J. Clin. Pharmacol. 83, 2242–2248. 10.1111/bcp.13329 28503781 PMC5595971

[B14] Schmidt L. E. Dalhoff K. (2002). Food-drug interactions. Drugs 62, 1481–1502. 10.2165/00003495-200262100-00005 12093316

[B15] Shen Z. Lee C. A. Valdez S. Yang X. Wilson D. M. Flanagan T. (2019). Effects of food and antacids on pharmacokinetics and pharmacodynamics of lesinurad, a selective urate reabsorption inhibitor. Clin. Pharmacol. Drug Dev. 8, 647–656. 10.1002/cpdd.663 30748125

[B16] Shi J. G. Chen X. Lee F. Emm T. Scherle P. A. Lo Y. (2014). The pharmacokinetics, pharmacodynamics, and safety of baricitinib, an oral JAK 1/2 inhibitor, in healthy volunteers. J. Clin. Pharmacol. 54, 1354–1361. 10.1002/jcph.354 24965573

[B17] Shi J. G. Chen X. McGee R. F. Landman R. R. Emm T. Lo Y. (2011). The pharmacokinetics, pharmacodynamics, and safety of orally dosed INCB018424 phosphate in healthy volunteers. J. Clin. Pharmacol. 51, 1644–1654. 10.1177/0091270010389469 21257798

[B18] Shibata M. Toyoshima J. Kaneko Y. Oda K. Kiyota T. Kambayashi A. (2021). The bioequivalence of two peficitinib formulations, and the effect of food on the pharmacokinetics of peficitinib: Two-way crossover studies of a single dose of 150 mg peficitinib in healthy volunteers. Clin. Pharmacol. Drug Dev. 10, 283–290. 10.1002/cpdd.843 32618438 PMC7984322

[B19] Singh B. N. (1999). Effects of food on clinical pharmacokinetics. Clin. Pharmacokinet. 37, 213–255. 10.2165/00003088-199937030-00003 10511919

[B20] Sparks J. A. (2019). Rheumatoid arthritis. Rheum. Arthritis. Ann. Intern. Med 170, ITC1-ITC16. ITC16. 10.7326/AITC201901010 30596879

[B21] Sun L. McDonnell D. Liu J. von Moltke L. (2019). Effect of food on the pharmacokinetics of a combination of olanzapine and samidorphan. Clin. Pharmacol. Drug Dev. 8, 503–510. 10.1002/cpdd.688 30921503

[B22] Taylor P. C. Keystone E. C. van der Heijde D. Weinblatt M. E. Del Carmen Morales L. Reyes Gonzaga J. (2017). Baricitinib versus placebo or adalimumab in rheumatoid arthritis. N. Engl. J. Med. 376, 652–662. 10.1056/NEJMoa1608345 28199814

[B23] US Department of Health and Human Services, Food and Drug Administration, Center for Drug Evaluation and Research (CDER) (2022). Assessing the effects of food on drugs in INDs and NDAs-clinical Pharmacology considerations guidance for industry. https://www.fda.gov/media/121313/download.

